# Hypercalcemia, an Important Puzzle Piece in Uncommon Onset Pediatric Sarcoidosis—A Case Report and a Review of the Literature

**DOI:** 10.3389/fped.2020.00497

**Published:** 2020-08-26

**Authors:** Cristina Oana Mărginean, Lorena Elena Meliţ, Gabriel Grigorescu, Claudiu Puiac, Iunius Simu

**Affiliations:** ^1^Department of Pediatrics, George Emil Palade University of Medicine, Pharmacy, Science and Technology of Târgu Mureș, Târgu Mureș, Romania; ^2^Pneumology Clinic, County Emergency Hospital Târgu Mureș, Târgu Mureș, Romania; ^3^Department of Emergency Medicine, George Emil Palade University of Medicine, Pharmacy, Science and Technology of Târgu Mureș, Târgu Mureș, Romania; ^4^Department of Radiology, George Emil Palade University of Medicine, Pharmacy, Science and Technology of Târgu Mureș, Târgu Mureș, Romania

**Keywords:** sarcoidosis, children, hypercalcemia, renal failure, uncommon onset

## Abstract

Sarcoidosis (SD) is a systemic granulomatous condition that is especially encountered in young adults and rarely in children, affecting predominantly the lungs and lymph nodes. We report the case of a 14-year-old teenage boy admitted to our clinic for nausea, vomiting, and weight loss. Clinical examination at the time of admission revealed malaise, pallor, and abdominal tenderness in the epigastric area at palpation. Laboratory tests revealed an elevated level of hemoglobin, mild thrombocytosis, increased erythrocyte sedimentation rate, and a mild increase in creatinine and urea levels along with hypercalcemia. An abdominal ultrasound revealed a right ectopic kidney, whereas the upper digestive endoscopy showed intense hyperemia and edema of the gastric mucosa. Thoracic computed tomography scan revealed giant hilar and mediastinal lymphadenopathy, along with multiple micronodules within the lung parenchyma and ground-glass aspect. The level of angiotensin-converting enzyme was high, parathormone was normal, and vitamin D level was low. Pathological examination of the bronchial, mediastinal, and lung biopsies established the diagnosis of SD. We administered oral corticosteroids for 2 months with outstandingly favorable outcome and no signs of recurrence 6 months after the cessation of the therapy.

## Introduction

Sarcoidosis (SD) or Besnier–Boeck–Schaumann disease was described for the first time in seventieth century as a systemic granulomatous condition of the lungs and lymph nodes (1). It is mainly a disease of the adults, with a peak onset between 25 and 45 years of age and with an incidence of ~5–45 in 100,000 adults (2), whereas it is almost 10 times less common in children (3). Even though its pathogenesis remains unknown, the current hypothesis involves an association between genetic predisposition and the environmental factors that demonstrate the ability to trigger or enhance the inflammatory and granulomatous processes (3).

The patients with SD express a wide spectrum of clinical manifestations, ranging from a subclinical form affecting only two organs to a life-threatening multiorgan dysfunction (4). The most common symptoms reported in children were related to lung and mediastinal involvement; however, extrarespiratory manifestations were also frequently observed, such as eye impairment, hepatomegaly, splenomegaly, skin anomalies, peripheral lymphadenopathy, or arthralgia (3, 5). Laboratory tests are not usually reliable for the diagnosis because they are not specific. Nevertheless, certain laboratory findings may be associated with SD in children, such as elevated erythrocyte sedimentation rate, anemia, hypergammaglobulinemia, lymphocytosis or lymphopenia, thrombocytosis, increased levels of transaminases and serum angiotensin-converting enzyme (ACE), and hypercalcemia (3), which also suggest other infectious pathologies (6). The assessment of renal function is also essential because hypercalcemia could lead to renal insufficiency or nephrolithiasis (7, 8). Multiple factors contribute to the development of hypercalcemia in SD patients, such as the upregulation of 1-α-hydroxylase activity within the granulomas of SD patients that leads to an increased conversion of 25-(OH) vitamin D_3_ into 1,25-(OH)_2_ vitamin D_3_, parathyroid adenomas, or supplementation of calcium in patients receiving chronic glucocorticoids (9). Nevertheless, normal levels of both forms of vitamin D were observed in hypercalcemic SD patients, which suggested that other mechanisms could also contribute to elevated serum calcium levels in these patients (10).

In SD children with respiratory symptoms, the first imaging investigation is chest radiography, which either can reveal hilar and mediastinal lymphadenopathies or lung fibrosis, or can be normal. Computed tomography (CT) is more sensitive and specific for mediastinal and lung involvement because it can highlight multiple imaging aspects suggestive of SD, such as interstitial lung disease, micronodules involving pleural surfaces, fissures, and peribronchovascular areas, perilymphatic nodules, ground-glass opacities, thickened septa, or alveolar consolidation (3). Flexible bronchoscopy using bronchoalveolar lavage is also reliable in assessing lung involvement. Nevertheless, pathological examination of the biopsied organs that reveal non-caseating granulomas remains the gold standard for SD diagnosis. Lung tissue biopsies could be obtained through wedge transbronchial lung biopsy; however, surgical biopsies are also a potential alternative (3). Other commonly biopsied organs in SD patients are peripheral or mediastinal lymph nodes and skin lesions (11).

Treatment may not be necessary for SD patients who present only hilar lymphadenopathy without parenchymal lung inflammation (12). Moreover, in children with SD, there is a lack of standardized therapeutic protocols, and they are usually treated in a similar manner as adults. Thus, administration of corticosteroids remains the most common therapeutic approach with significant improvement within 6 months. Considering the unproven favorable long-term outcome for lung impairment, other drugs/agents may also be useful, such as methotrexate, cytotoxic agents, or antimalarials (13). The prediction of SD prognosis in children is difficult owing to its rarity at these ages; however, the mortality rates are estimated to vary between 1 and 5% (14).

The aim of this case report was to underline the uncommon onset of SD in a teenage boy due to associated hypercalcemia.

Written informed consent was obtained from the patient's mother prior to the publication of this case report.

## Case Report

### Presenting Concerns

A 14-year-old boy who presented with nausea, vomiting, and weight loss (~6 kg within 1 month) was admitted to the Department of Pediatric Gastroenterology with the suspicion of acute gastritis. The patient denied previous vitamin D supplementation or sun exposure.

### Clinical Findings

Clinical examination at the time of admission revealed malaise, pallor, and abdominal tenderness in the epigastric area at palpation. The patient weighed 50 kg.

### Diagnostic Focus and Assessment

**Figure 1:**
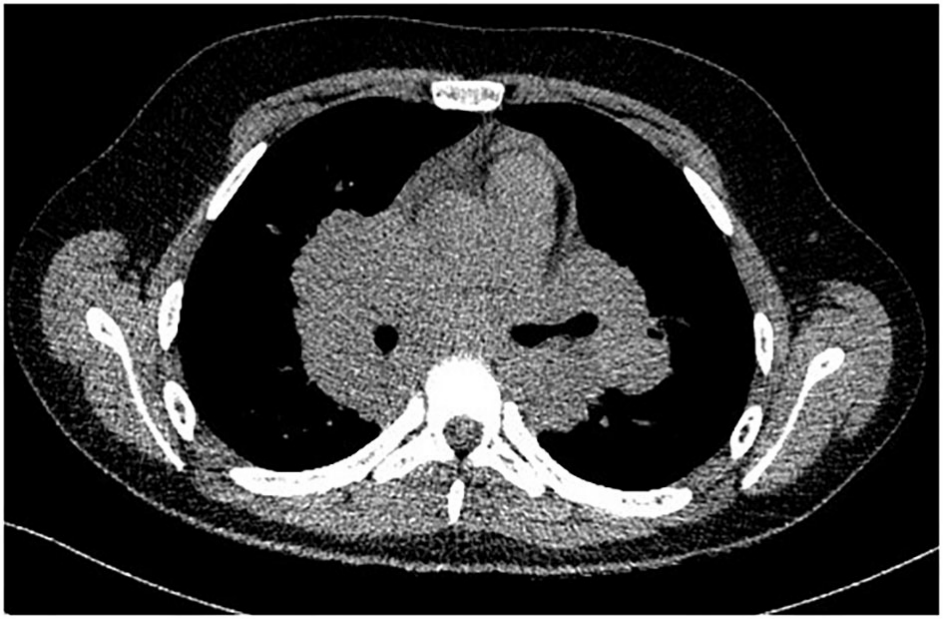
CT aspect of mediastinal lymphadenopathies.

**Figure 2:**
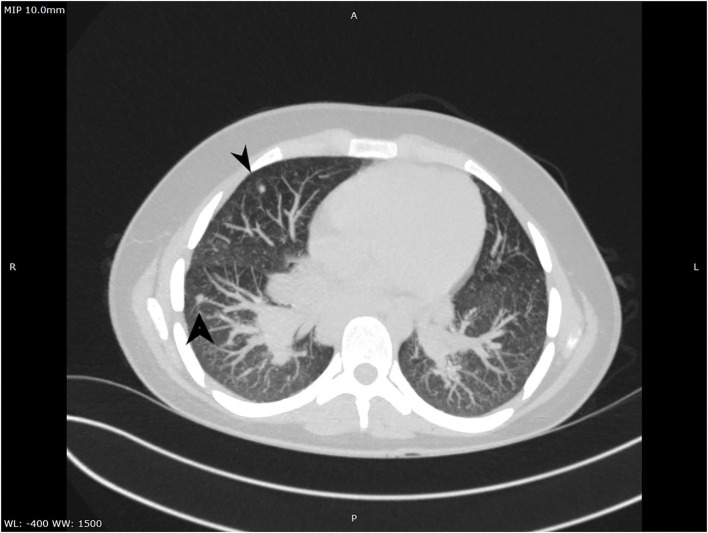
CT aspect of lung micronodules.

**Figure 3:**
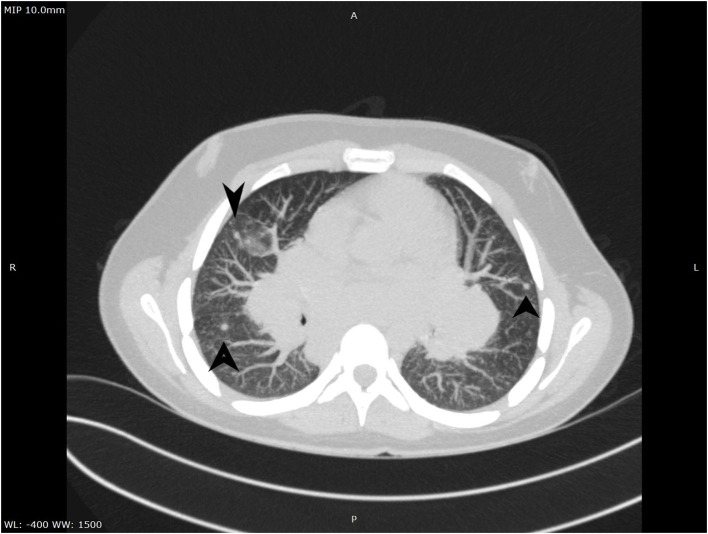
Ground-glass aspect on CT.

A complete blood count revealed an elevated level of hemoglobin (15.7 g/dL) and mild thrombocytosis (518,000/μL) along with an elevated erythrocyte sedimentation rate (23 mm/h). Biochemical blood analysis showed high levels of creatinine (156.5 μmol/L) and urea (22.35 mmol/L), along with hypercalcemia (total serum calcium 3.66 mml/L, serum ionized calcium 1.77 mmol/L). A qualitative test performed from a urinary sample proved hypercalciuria, and the routine urine examination revealed microscopic hematuria (10 erythrocytes per field). Renal ultrasound revealed a right ectopic kidney without any associated pathological findings in terms of kidney morphology and structure. We consulted an endocrinologist, who ruled out any associated conditions. An upper digestive endoscopy demonstrated intense hyperemia and edema of the gastric mucosa, and pathological examination of the gastric biopsies established the diagnosis of *Helicobacter pylori*–induced gastritis. This was treated according to the standard guidelines, using double-antibiotic regimen (amoxicillin and clarithromycin) that influences proton pump inhibitors. The elevated levels of urea and creatinine were initially interpreted as signs of prolonged dehydration; however, despite proper intravenous fluid administration, they remained above the normal range. Thus, we decided to perform thoracic and abdominal CT. Thoracic CT revealed giant hilar and mediastinal lymphadenopathies (Figure 1), multiple micronodules (Figure 2) within the lung parenchyma, and ground-glass opacities (Figure 3), which raised the suspicion of SD, without ruling out tuberculosis or lymphoma, whereas the abdominal scan confirmed the ultrasound findings showing only a right ectopic kidney. We found an elevated level of ACE (244.3 U/L), normal level of parathormone (15.1 pg/mL), and decreased levels of 1,25-(OH)_2_ vitamin D (14.75 ng/mL). The cardiologic and ophthalmologic examinations were normal. Additional tests, such as peripheral smear, protein electrophoresis, human immunodeficiency virus serology, and serum copper were performed in order to rule out other conditions that evolve with lung and lymph node impairment. In order to confirm our suspicion of SD, a bronchoscopy with bronchial biopsy and open surgery with lung and mediastinal biopsies were performed because an endobronchial ultrasound approach was not possible. The pulmonary function tests performed prior to the bronchoscopy were within normal ranges. The pathological examination revealed multiple non-caseating granulomas in all the biopsy specimens, which established the diagnosis of SD.

### Therapeutic Focus and Assessment

We initiated the treatment with oral administration of prednisone at a loading dose of 1 mg/kg per day, that is, 50 mg/d, with tapering doses for 2 months, while considering the improvement in the patients' symptoms after the first week of therapy and findings of the blood tests after ~2 weeks.

### Follow-Up and Outcome

**Figure 4:**
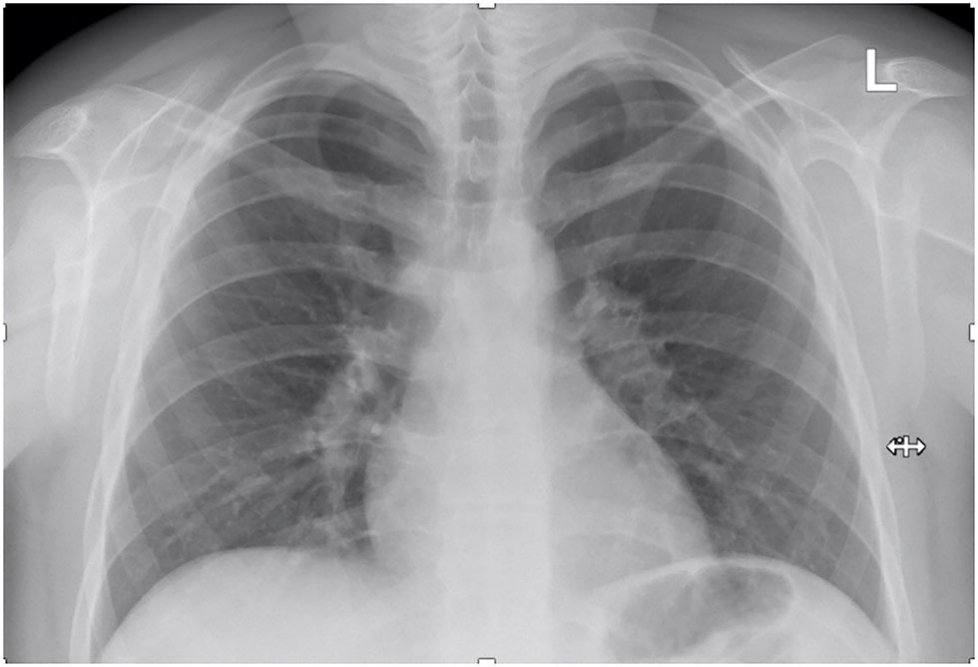
Follow-up chest X-ray.

The patient's follow-up for ~6 months after the cessation of corticosteroid administration revealed favorable clinical outcome with normal results of all blood tests, inclusively renal function parameters, and normal ACE levels. Moreover, the chest X-ray showed no enlarged lymph nodes and a normal aspect of the lung parenchyma (Figure 4).

## Discussion

SD rarely occurs in children; it primarily affects young adults (2). Nevertheless, here, we describe a case of SD in a 14-year-old teenage boy with lung and mediastinal involvement. Respiratory symptoms are most commonly observed at onset in the patients with this type of involvement (3). Nevertheless, our patient presented with nausea, vomiting, and weight loss most likely due to *H. pylori*–induced gastritis.

SD patients frequently exhibit anemia, thrombocytosis, lymphocytosis, or lymphopenia (3). In our patient, we encountered only thrombocytosis and, contrary to the general observation, an increased level of hemoglobin. As observed for our patient, erythrocyte sedimentation rate may also be elevated in SD patients as a sign of mild inflammation similar to that observed in other conditions (15–17). Among the other altered laboratory parameters previously reported in SD children, including hypergammaglobulinemia, elevated transaminases, increased ACE, and hypercalcemia, our patient exhibited elevated levels of ACE and hypercalcemia. Moreover, he exhibited impairment of renal function as reflected by elevated creatinine and urea. Despite their initial indications as dehydration signs, the increase in renal function parameters was most likely caused by SD-associated hypercalcemia. Other studies stated that ~30% of the patients diagnosed with SD exhibit asymptomatic hypercalcemia, although nephrocalcinosis is rarely observed in these patients (11). The imaging assessment excluded nephrocalcinosis in our patient, but the increase in urea and creatinine levels was strongly related to hypercalcemia. Moreover, Milman et al. (18) stated that hypercalcemia, and cutaneous sarcoid lesions and generalized lymphadenopathy are markers of poor prognosis in patients with SD. Despite the fact that our patient presented hypercalcemia, the outcome was favorable after ~2 weeks from the initiation of corticosteroids when the serum calcium levels became normal. Nevertheless, we did not find any skin lesions, and the lymphadenopathies were localized. SD-associated renal changes have previously been classified into three main types: primary, parenchymatous, or granulomatous SD; glomerulonephritic changes resembling collagen diseases or nephrotic syndrome; and hypercalcemic renal damage, which occurs most frequently (19). Thus, patients with SD-associated hypercalcemia may present a wide spectrum of signs and symptoms, including renal colic (due to recurrent kidney stones), mental disorders, loss of appetite, constipation, diffuse abdominal pain, gastric ulcers, myopathy, or neuromuscular symptoms (19). The authors described two cases of SD-associated hypercalcemia and uremia in two adult patients with favorable outcome after administration of corticosteroids, which demonstrated the high efficacy of steroid therapy in these cases (19). Nevertheless, the reports in children are scarce.

ACE is thought to be produced by both SD granulomas and lung endothelium (20). Although the levels of this enzyme have been shown to be elevated in SD patients, its sensitivity and specificity as a diagnostic marker may vary between 41 and 58% and between 84 and 90%, respectively (21). Regardless of these discordant reports, elevated ACE levels may be associated with disease activity and also represent efficient marker of treatment efficacy (22). A recent study performed on 119 adult SD patients, which aimed to establish the role of ACE, showed that elevated ACE levels are associated with high serum levels of ionized calcium (23). Similarly, in our case, we noticed elevated levels of both ACE and serum levels of ionized calcium that became normal after treatment, suggesting indeed that ACE might be a potential therapeutic marker.

The communications skills of a physician are essential for a proper anamnesis in order to rule out other potential risk factors or associated conditions (24). Chest X-ray and pulmonary function tests are important for the follow-up of SD patients; however, initial small lesions may not be observed using these diagnostic tools (14). Thus, CT represents the gold standard in diagnosing SD in pediatric patients that require further investigations (25). In our case, thoracic CT revealed giant hilar and mediastinal lymphadenopathies, multiple micronodules within the lung parenchyma, and ground-glass opacities, all suggestive of SD. Despite all the information provided by anamnesis, clinical examination, and imaging assessment, only a pathological examination could confirm SD diagnosis (26). In our patient, non-caseating granulomas were identified in all biopsy specimens. Because the transbronchial approach was unsuccessful, we were forced to perform an open surgery for mediastinal and lung biopsies in order to confirm the diagnosis and to exclude the possibility of other pathologies (27). Thus, other similar granulomatous disorders include Blau syndrome, autoimmune lymphoproliferative syndrome, or Crohn disease. Blau syndrome is defined by a triad consisting in granulomatous dermatitis, arthritis, and uveitis, occurring commonly during early childhood (28). Contrariwise, our patient presented no signs of dermatitis, arthritis, and uveitis. Wegener granulomatosis, an idiopathic inflammatory disorder, is characterized by necrotizing granulomatous inflammation and pauci-immune small-vessel vasculitis affecting the upper and lower respiratory tracts and kidneys, and the diagnosis is usually established based on histopathological examination (29). Nevertheless, our patient presented no signs of vasculitis involving the upper and lower respiratory tracts or kidney, and the histopathological examination confirmed the diagnosis of SD. An uncommon condition that must be taken into account in patients with lymphadenopathy is the autoimmune lymphoproliferative syndrome defined also by splenomegaly and autoimmune cytopenia (30), these were absent in our case. Chron's disease is another granulomatous condition affecting the gastrointestinal tract (31), which was ruled out in our case based on the upper digestive endoscopy and pathological examination of the gastric mucosa biopsy. Except all these rare conditions, all the remaining common infectious or inflammatory disorders, including tuberculosis, were ruled out based on the pathological examination.

Despite possible spontaneous remission, visual observation is not an efficient management strategy in case of pediatric patients (3). Oral or intravenous administration of corticosteroids is primarily efficient in the management of SD children, especially in those with SD-associated hypercalcemia (3). In previously reported cases, the duration of treatment varied from 1 month to 23 years (5, 11, 32, 33). We decided to cease corticosteroids after 2 months owing to the favorable outcome of our patient after 2 weeks of treatment. The prognosis of SD in children depends on the onset age, disease extension, and response to corticosteroids (3). We hope that the patient we presented would have a favorable outcome because of his older onset age, disease limitation to lung and mediastinum, and rapid favorable response to corticosteroids. Nevertheless, prognostic factors still remain unclear in children (3).

## Conclusions

SD occurs rarely in children. Even though spontaneous remission is possible in patients with SD, corticosteroids result in an outstandingly favorable evolution in SD children with primarily mediastinal and lung involvement associated with hypercalcemia. SD in children remains an “exclusive diagnosis;” however, awareness of the physician is vital for its early diagnosis.

## Ethics Statement

Written informed consent was obtained from the legal guardian, for the publication of any potentially identifiable images or data included in this article.

## Author Contributions

CM and LM conceptualized and designed the study, drafted the initial manuscript, and reviewed and revised the manuscript. CP, GG, and IS designed the data collection instruments, collected data, carried out the initial analyses, and reviewed and revised the manuscript. All authors approved the final manuscript as submitted and agree to be accountable for all aspects of the work.

## Conflict of Interest

The authors declare that the research was conducted in the absence of any commercial or financial relationships that could be construed as a potential conflict of interest.

## Abbreviations

ACE: angiotensin-converting enzyme
CT: computed tomography
SD: sarcoidosis.
